# Regulation of endogenous cardiomyocyte proliferation: The known unknowns

**DOI:** 10.1016/j.yjmcc.2023.04.001

**Published:** 2023-06

**Authors:** Ilaria Secco, Mauro Giacca

**Affiliations:** School of Cardiovascular and Metabolic Medicine & Sciences and British Heart Foundation Centre of Research Excellence, King's College London, London, United Kingdom

## Abstract

Myocardial regeneration in patients with cardiac damage is a long-sought goal of clinical medicine. In animal species in which regeneration occurs spontaneously, as well as in neonatal mammals, regeneration occurs through the proliferation of differentiated cardiomyocytes, which re-enter the cell cycle and proliferate. Hence, the reprogramming of the replicative potential of cardiomyocytes is an achievable goal, provided that the mechanisms that regulate this process are understood. Cardiomyocyte proliferation is under the control of a series of signal transduction pathways that connect extracellular cues to the activation of specific gene transcriptional programmes, eventually leading to the activation of the cell cycle. Both coding and non-coding RNAs (in particular, microRNAs) are involved in this regulation. The available information can be exploited for therapeutic purposes, provided that a series of conceptual and technical barriers are overcome. A major obstacle remains the delivery of pro-regenerative factors specifically to the heart. Improvements in the design of AAV vectors to enhance their cardiotropism and efficacy or, alternatively, the development of non-viral methods for nucleic acid delivery in cardiomyocytes are among the challenges ahead to progress cardiac regenerative therapies towards clinical application.

## Highlights

• Cardiac regeneration through the reprogramming of the endogenous cardiomyocyte proliferation potential is an attainable goal
• Cardiomyocyte proliferation is controlled by extracellular cues through at least four signal transduction pathways
• Administration of both protein coding genes and non coding RNAs can stimulate cardiomyocyte proliferation
• Significant advances are needed to deliver the pro-proliferative nucleic acids to the heart using viral on non viral vectors

## Graphical abstract

**Figure:**
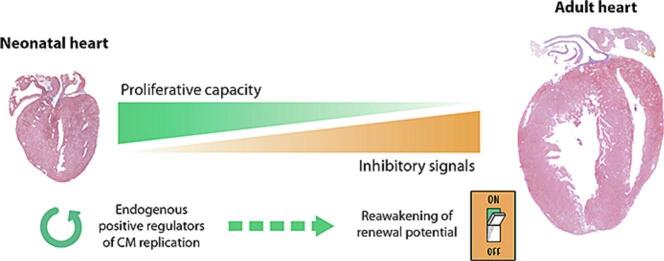

## 1 Introduction

Heart failure is common, deadly, and expensive. This condition now affects 8.52 per 1000 individuals from the general population [1], has a mortality estimated at 40% at only 4 years from diagnosis [2], and absorbs 2–3% of national health expenditures in high-income countries, with a projection to more than double in the next 20 years [3]. Heart failure is a common consequence of cardiac damage. This can be sudden, as after myocardial infarction when up to 25% of cardiac cells in the left ventricle can die, some of which immediately and others in the first several hours after acute ischemia [4], or chronic, as it accompanies virtually all the diseases that affect the myocardium. Loss of cardiomyocytes occurs in hypertensive cardiac disease [5], aortic stenosis [6], or viral myocarditis [7]. Cardiomyocyte death also accompanies all forms of inherited cardiac conditions, ranging from Danon cardiomyopathy [8] to arrhythmogenic cardiomyopathy [9]. Finally, cardiac cell loss occurs during perioperative myocardial injury and reperfusion [10] and because of antineoplastic therapy, in particular using anthracyclines [11]. The loss of functional contractile mass in the heart leads to decreased cardiac output, with consequently impaired capability of facing hemodynamic challenges, and eventually leading to heart failure with reduced ejection fraction.

This sudden or progressive loss of cells is not accompanied by significant new cardiomyocyte generation after birth. The regenerative capacity of the normal adult human heart was estimated, through ^14^C‑carbon dating, to be less than 50% renewal in a 70-year lifetime [12]. This estimate is consistent with measurements in mice obtained by analysing DNA synthesis [13] or using imaging mass spectrometry [14]. This minimal residual regenerative capacity is well beyond what would be required to compensate for pathological cardiomyocyte loss. In sharp contrast to other species, including fish and amphibians in which cardiac regeneration after damage can occur throughout life [15,16], mammalian cardiomyocytes stop dividing after birth to never re-enter the cell cycle in a significant manner in adulthood.

The repair of damaged hearts thus remains a holy grail in clinical medicine. One appealing possibility to achieve cardiac regeneration is to stimulate the proliferation of endogenous cardiomyocytes, as opposed to the implantation of cardiac progenitor cells [17] or differentiated cardiomyocytes [18], or the reprogramming of other cell types into cardiomyocytes [19,20]. Activating the cell cycle in cardiomyocytes, however, requires a thorough understanding of the molecular reasons why these cells do not proliferate spontaneously after myocardial damage, the identification of factors that can be used for therapeutic purposes, and the development of methods for their effective administration. Here, we review our current knowledge about the molecules and pathways that are known to regulate cardiomyocyte proliferation. We also highlight the many unknowns that still hamper the successful generation of a new class of medicines that could promote the proliferation of endogenous cardiomyocytes to achieve cardiac regeneration in the clinic.

## 2 Molecular regulation of cardiomyocyte proliferation

**Fig. 1:**
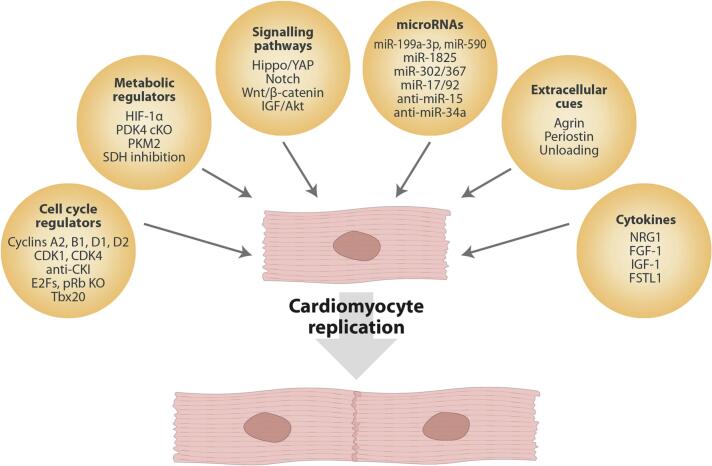
Extracellular and intracellular regulators of cardiomyocyte replication. The cartoon groups the main categories of molecules and pathways involved in the regulation of cardiomyocyte proliferation. See text for discussion and references. KO: knockout; cKO: conditional knockout.

Over the last few years, a number of molecules and pathways have been reported to regulate cardiomyocyte proliferation acting both inside and outside the cells. The molecules acting intracellularly include cell cycle and metabolic regulators, microRNAs and activators of at least 4 different signalling pathways. The extracellular cues include factors acting from the extracellular matrix to the cardiomyocyte sarcolemma and a few cytokines. These are visually summarised in Fig. 1 and discussed in the following sections.

1) **Regulators of the cardiomyocyte cell cycle.**

The arrest of cardiomyocyte proliferation after birth is contextual to the downregulation of essential cell cycle factors and the upregulation of cell cycle inhibitors. Cell cycle activators, such as cyclins, cyclin-dependent kinases (CDKs), c-Myc oncogene and E2F transcription factors are repressed [13,21,22], whereas the levels of negative cell-cycle regulators, such as p21, p27, retinoblastoma protein (Rb), and cyclin-dependent kinase inhibitors (CKIs) are increased [[23], [24], [25]]. The above cited studies on individual factors have been more recently corroborated by global transcriptomic and epigenetic analyses in both mice [26,27] and humans [28], which have confirmed strong repression of cell cycle genes in adult cardiomyocytes.

**Fig. 2:**
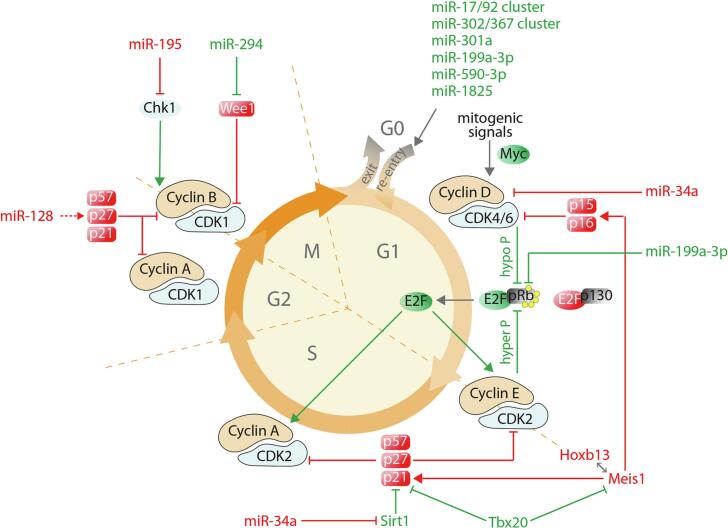
Molecular switches for cardiomyocyte cell cycle regulation. The factors that were experimentally proven to control cell cycle entry or progression in cardiomyocytes are shown. Green: positive regulators, red: negative regulators. See text for descriptions. (For interpretation of the references to colour in this figure legend, the reader is referred to the web version of this article.)

Similar to other replicating cells in the body, most of the regulation of cardiomyocyte proliferation is exerted in the G1 phase of the cell cycle (Fig. 2). In early G1, E2F proteins activate transcription of S-phase Cyclin A and of other genes required for DNA synthesis. In response to mitogenic stimulation, Cyclin D-CDK4/6 and later Cyclin *E*-CDK2 complexes phosphorylate and inactivate the E2F repressor Rb. E2F factors also control the transcription of their own genes and the G1/S-phase Cyclin E, resulting in a positive cross-regulation. As an upstream regulator of Cyclin E and Cyclin A, overexpression of E2F1 in the heart stimulates DNA synthesis but results in an increased rate of cell death [29,30], while overexpression of E2F2 in adult mice can modestly increase cardiomyocyte mitosis and cytokinesis [31]. In a consistent manner, inducible knockout of Rb and p130 (which results in repressive histone methylation of E2F-dependent genes) decreases the level of heterochromatin and re-activates cycle genes, leading to proliferation [32].

By triggering progression through the G1 restriction point, Cyclin D-CDK4/CDK6 and Cyclin *E*-CDK2 complexes are responsible for the initiation of the S phase. Cardiomyocyte-restricted Cyclin D1 overexpression increases DNA synthesis without progression through cytokinesis, resulting in augmented multinucleation [33,34] whereas Cyclin D2 overexpression induces DNA synthesis and regenerative growth after myocardial infarction in mice [35] and reduces myocardial remodelling and dysfunction after pressure overload [36]. Cyclin D2 overexpression in hiPSC-derived-cardiomyocytes promotes cell cycle progression and telomerase activity, increasing the number of engrafted cells after transplantation in both mouse [37] and pig [38] infarcts and exerting beneficial effects on scar reduction and angiogenesis. Interestingly, Cyclin D2-overexpressing hiPSC-CMs also promote proliferation when the recipient porcine cardiomyocytes are close to the graft, through the secretion of exosomes enriched in pro-proliferative miRNA-302 and -373 cluster microRNAs [38].

S phase progression is assisted by the Cyclin A-CDK2 complex, which stimulates chromosome duplication and helps in controlling early mitotic events. Moreover, Cyclin A, this time in concert with CDK1, stimulates the entry in mitosis at the G2/M checkpoint; the Cyclin B-CDK1 complex also has a similar role. Cardiomyocyte-restricted overexpression of Cyclin A2 induces mitosis both in non-injured [39] and infarcted mouse hearts [40]. Delivery of an adenoviral vector expressing Cycling A2 to infarcted rats induces cardiomyocyte cell cycle activation and increases myofilament density in the infarct border zone, while improving cardiac function [41]. A similar effect is obtained with a Cyclin A2 expressing adenoviral vector administrated to the porcine peri-infarct zone, including amelioration of cardiac function, decreased fibrosis and increased cardiomyocyte density [42]. Overexpression of Cyclin B1 and a constitutively active CDK1 in adult rat cardiomyocytes also stimulates cell division in vitro [43].

Cyclin-dependent kinase activities are modulated by interactions with Cyclins and Cyclin-dependent kinase inhibitors (CKIs). Mice knock out for the p21 or p27 CKIs show failure in cell cycle exit at G1-phase and endoreduplication without successful cell division [25]. AAV9-mediated cardiac delivery of the histone deacetylase Sirt1, which has multiple targets including p21, causes p21 destabilization and increases mitotic markers [44]. Along the same line, the triple knockdown of p21, p27 and p57 induces both neonatal and adult cardiomyocytes to enter S-phase and complete mitosis in vitro without overt DNA damage and aberrant mitosis. A subset of the treated cells also progresses through cytokinesis [45].

Meis1, which belongs to the TALE (three amino acid loop extension) family of homeodomain transcription factors, is one of the central regulators of cardiac differentiation. Its expression increases postnatally in the heart where it activates transcription of p15, p16 and p21, which in turn switch off CDK6, 4 and 2 and arrest the cell cycle. Conditional knockout mice for Meis1 show extended postnatal proliferation and reactivation of mitosis in the adult heart, whereas overexpression of Meis1 impairs neonatal cardiomyocyte proliferation and regeneration [46]. Cardiomyocyte-specific deletion of Hoxb13, a Meis1 cofactor, extends the postnatal window of cardiomyocyte proliferation and reactivates the cardiomyocyte cell cycle in the adult heart. Moreover, adult Meis1-Hoxb13 double-knockout hearts display widespread cardiomyocyte mitosis, sarcomere disassembly and improved left ventricular systolic function following myocardial infarction [47].

Another transcription factor that interferes with the same cell cycle regulatory pathways is Tbx20, which is essential for cardiomyocyte proliferation during embryonic development. Overexpression of Tbx20 in adult cardiomyocytes promotes proliferation [48] due to the induction of Cyclin D1, E1, and IGF-1 and repression of p21 and Meis1. Notably, Tbx20 increases the expression of the foetal contractile proteins ssTnI (skeletal muscle, slow troponin I type 1) and β-MHC (beta-myosin heavy chain). As a result, after myocardial infarction, the presence of Tbx20 preserves cardiac function and improves survival [49]. Of potential interest, Tbx20 was also reported to improve cardiac reprogramming induced by a cocktail of factors consisting of MEF2C, GATA4, TBX5 and miR-133 [50].

Together, the above-mentioned studies provide proof-of-principle evidence that the cardiomyocyte cell cycle can be stimulated by the exogenous administration of positive cell cycle regulators, or the inhibition of those that exert a negative role. However, the impact of such treatments only seems to arise in a very small percentage of cells. This likely implies that a single regulator is not sufficient and that multiple pathways must be turned on to breach the diverse walls that block cardiomyocyte cell division. An attempt at multifactorial targeting was made through a combinatorial screening of the most differentially expressed cell cycle regulators between embryonic and adult mouse hearts, followed by the administration of a cocktail of four factors (Cyclin D-CDK4 and Cyclin B-CDK1) after myocardial infarction in mice [51]. The results showed significant improvement in cardiac function after myocardial infarction.

Whether all cardiomyocytes can re-enter the cell cycle or only a subset of cardiac cells participates in the regenerative process is still unclear. Single nucleus RNA sequence analysis indicates that only a specific subgroup of cardiomyocytes becomes proliferative after myocardial injury in neonate mice, which disappears as the heart loses the ability to regenerate [52]. Consistent with this notion, the above-cited study based on the administration of four cell cycle regulators also showed that only 15%–20% of adult cardiomyocytes expressing these factors underwent stable cell division, suggesting specificity in the cells that are capable of proliferation.

An important problem in the field is to understand the molecular pathways that regulate entry into the S-phase, nuclear division and cell cytokinesis, as the three processes appear to be loosely correlated given that cardiomyocytes are often polypoid and/or multinucleated. Rodents are born with almost all cardiomyocytes that are mononucleated and diploid [53]. By the second week of life, however, binucleation occurs in more than 90% of cells [54,55] secondary to a burst in cell cycle activity that takes place during the first days of life. Binucleation occurs when a cell completes mitosis without a division of its cytoplasm (cytokinesis). Since cardiomyocyte cell cycle activity is commonly reported to be higher in mononucleated cells [14,56,57], the process of multinucleation may account for the dramatic reduction in cardiac regeneration.

In humans, the extent of multinucleation is much lower (around 10–25%) and is established during the first year of life [58], as most cardiomyocytes undergo a final round of DNA replication without nuclear or cellular division resulting in mononucleated cells with tetraploid DNA content. Studies on heart tissues from different ages have revealed that the process of polyploidization in humans takes about 20 years, a timeframe in which the percentage of polyploid cardiomyocytes increases significantly [59].

The mechanisms that drive polyploidy in favour of binucleation in cardiomyocytes from different species remain unclear. Both processes appear to occur as a response to different cellular stressors, as further discussed below. Similarities with other cell types, such as hepatocytes and keratinocytes, suggest that multinucleation and polyploidization could be ways to increase longevity and tolerance to genomic stress and apoptosis, as well as to sustain high levels of mRNA and protein synthesis [60]. More specific for the heart, multinucleation and polyploidy appear to be efficient strategies to increase cell size and organ mass without disrupting cellular and tissue structure, thus preserving contraction function. Analyses from both fixed tissue samples [61] and cell suspensions [62] show that multinucleated cardiomyocytes have a larger volume compared to mononucleated cells. These considerations appear to be of relevance in light of the therapies that aim to increase endogenous cardiac regeneration. As the ultimate goal of these therapies is to increase heart muscularization, it could result of minor functional relevance whether this is achieved through mononuclear cell proliferation or increase in muscle fibre size by multinucleation and/or polyploidisation.

2) **Signal transduction pathways that regulate cardiomyocyte proliferation.**

**Fig. 3:**
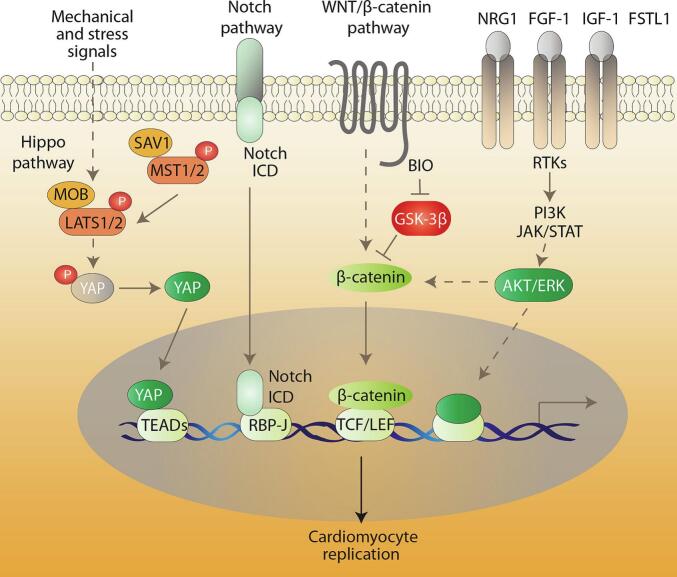
Main signal transduction pathways leading to cardiomyocyte replication. All four pathways (inhibition of Hippo, activation of Notch or of β-catenin, or receptor-mediated activation of PI3K/AKT) activate a transcriptional program that leads to cell replication. In the cases of Hippo, Notch and Wnt/β -catenin, this programme is triggered by nuclear translocation of the transcriptional co-activators YAP, Notch Intracellular Domain (ICD) and β -catenin respectively, each of which bind to its cognate specific transcription factor (members of the TEAD family, RBP-J and TCT/LEF respectively).

The cardiomyocyte cell cycle is regulated by a variety of different signalling pathways, most of which are involved in heart development. Interest for these regulatory mechanisms focuses on both understanding the biology of cardiomyogenesis and exploiting this knowledge to stimulate regeneration. There are at least four signal transduction pathways that are involved in the regulation of cardiomyocyte proliferation - the Hippo, Wnt/β-catenin, Notch and PI3K/AKT pathways (Fig. 3).

The Hippo pathway plays a pivotal role in the regulation of several organ size and growth, controlling cell proliferation, apoptosis and differentiation. Activation of this pathway by a protein kinase cascade leads to the phosphorylation and inactivation of the transcriptional coactivator YAP (Yes-associated protein). In its active form, YAP translocates into the nucleus where it associates with the transcriptional enhanced associate domain (TEAD) 1–4 transcription factors to drive gene expression and eventually stimulate cell proliferation. Deletion of YAP during development results in myocardial hypoplasia and early embryonic lethality [63,64]. Transgenic mice overexpressing activated (de-phosphorylated) YAP repair myocardial injury through regeneration instead of fibrosis [65,66]. In a consistent manner, mice lacking Salvador, a scaffolding protein for Mst inactivating kinases, show enhanced regeneration after cardiac apex resection or myocardial infarction [67,68]. Hippo signalling intertwines with that of many other signalling pathways. Expression of the IGF-1 receptor is increased in hearts expressing the constitutive form of YAP [64], while YAP/TEAD complexes associate with nuclear β-catenin at pro-proliferative Wnt target genes such as Sox2 and Snai2 [69]. Moreover, one of the YAP target genes is Pik3cb, which in turn activates the PI3K-AKT pathway discussed below [70].

Notch signalling controls cell fate specification and mesodermal commitment during development, regulating genes involved in proliferation, stem cell renewal, differentiation and apoptosis. In particular, Notch sustains the proliferation of immature cardiomyocytes during foetal and post-natal life [[71], [72], [73]]. Although its role in driving cardiac regeneration in adult zebrafish heart is well documented [74], the exogenous activation of Notch in adult rodent hearts is ineffective because of the suppressive epigenetic modifications at Notch-responsive promoters that occurs in adulthood [75].

The Wnt/β-catenin pathway not only is important for second heart field development [76], but also, more in general, because it controls cardiomyocyte cell proliferation, survival, apoptosis, adhesion and hypertrophy. In basal conditions, cytoplasmic β-catenin is the target of the β-catenin destruction complex, which is formed of Axin, adenomatous polyposis coli (APC), casein kinase 1 (CK1) and glycogen synthase kinase 3β (GSK-3β); CK1 and GSK-3β phosphorylate β-catenin, which is then recognised by the SCF-βTrCP E3 ubiquitin ligase and targeted for degradation by the proteasome. Activation of canonical Wnt signalling inhibits the destruction complex, the levels of β-catenin in the cytoplasm increase, and β-catenin is eventually translocated into the nucleus where it binds the T cell factor (TCF)/Lymphoid enhancer factor (LEF) family of transcription factors to drive a transcriptional programme that leads to cell proliferation [77]. Treatment of cardiomyocytes with an inhibitor of GSK3-β causes cell proliferation [78], while genetic ablation of GSK3-β in mice leads to cardiomyocyte hyperproliferation followed by hypertrophic cardiomyopathy [79]. The YAP and TAZ transcriptional cofactors in the Hippo pathway are also major regulators of β-catenin stability [80,81].

Finally, the PI-3-Kinase (PI3K)/AKT pathway is crucial to promote the growth and survival of several cell types, including cardiomyocytes. AKT inactivates pro-apoptotic proteins, activates Cyclin D1 and CDK2, inhibits p27 and regulates cell growth through mTOR activity (reviewed in ref. [82]). AKT can also inactivate GSK-3β ensuing in β-catenin stabilization [83]. PI3K is downstream various receptor tyrosine kinases, some of which have a fundamental role in cardiac development and cardiomyocyte proliferation. Among these, the epidermal growth factor receptors ErbB2 and ErbB4, expressed on the cardiomyocyte plasma membrane, bind Neuregulin-1 (NRG1) to sustain cardiomyocyte proliferation during embryogenesis. NRG1-induced cardiomyocyte proliferation diminishes after birth due to a reduction in ErbB2 expression, but exogenous activation of NRG-1/ErbB2–4 signalling induces differentiated and quiescent cardiomyocytes to re-enter the cell cycle and undergo karyokinesis in a PI3K/AKT dependent manner, also leading to cardiac regeneration in adult infarcted mice [84,85]. The extracellular matrix protein periostin, which is re-expressed in injured adult myocardium, has been reported to activate cardiomyocyte mitosis via integrins and PI3K and improve ventricular remodelling after myocardial infarction [86,87]. PI3K/AKT is also one of the pathways activated by fibroblast growth factor-1 (FGF-1), which, in combination with the inhibition of p38 kinase (a known negative regulator of the cell cycle), increases mitotic events in mice and rats, improving heart function after myocardial ischemia [88,89]. The insulin-like growth factor-1 (IGF-1) acts through PI3K/AKT signalling to inactivate GSK-3β, thus leading to the stabilization of the Wnt final effector β-catenin. Cardiac overexpression of IGF-1 in mice stimulates proliferation and prevents cell death and ventricular dilation after infarction [90]. There is evidence, albeit far from being completely elucidated, that AKT is involved in the transduction of the signal from follistatin-like-1 (FSTL1), a glycoprotein secreted by the epicardium. FSTL1 expression shifts to cardiomyocytes following myocardial infarction [91]{Wei, 2015 #498}. Only the epicardial-derived FSTL1 can sustain cardiac regeneration and repair after infarction in mice and pigs [91,92]. The cardiomyogenic function of FSTL1 has been linked to the absence of glycosylation in a specific site when synthesised in epicardial cells [93].

3) **Regulation of cardiomyocyte proliferation by microRNAs.**

Several microRNAs (miRNAs) that tune cardiomyocyte proliferation have been identified by both transcriptional profiling in pre- and post-natal hearts [94] and high throughput screenings of synthetic miRNA mimic libraries [95,96] (Fig. 2). The pro-proliferative miRNAs that were identified include the miR-17/92 cluster, which was initially discovered as a human oncogene promoting cell proliferation, but also exerts an indispensable role in the development of various organs, including the heart. Transgenic overexpression of miR-17/92 in cardiomyocytes is sufficient to induce proliferation in embryonic, postnatal, and adult hearts, where members of this cluster also protect cardiac tissue from myocardial infarction injury [97,98]. Another cluster of miRNAs that exerts a strong pro-proliferative effect on cardiomyocytes is the miR-302/367 cluster. These miRNAs are expressed during early embryogenesis and are important for cardiomyocyte proliferation during development. The miRNA members of the miR-302/367 cluster act by inhibiting upstream negative regulators of the Hippo pathway, namely Mst1, Lats2 and Mob1b. Reactivation of miR-302/367 postnatally in mice induces cardiac regeneration after myocardial infarction but leads to long-term cardiomyocyte dedifferentiation and dysfunction [99]. Other pro-proliferative miRNAs are human miR-199a-3p, miR-590-3p and miR-1825, which were initially identified in our laboratory through an in vitro functional screening for cardiomyocyte proliferation in rodents. These miRNAs also exert their activity in experimental models of myocardial infarction of both mice and pigs, inducing cardiac regeneration and markedly improving cardiac function one month after injury [95,100,101]. Two miRNAs that were identified by differential expression in the developing vs. the adult heart are miR-301 and miR-294. AAV9-mediated cardiac delivery of miR-301 in infarcted mouse hearts promotes regeneration and cardiac repair involving the PTEN/AKT/cyclin D1 pathway [102]. Instead, miR-294 represses Wee1 leading to increased activity of the Cyclin B1/CDK1 complex. When infarcted mice received a doxycycline-inducible AAV9-miR-294 vector, cardiac function improved due to cardiomyocyte re-entry into the cell cycle [103].

Other miRNAs act in an opposite manner by inhibiting cardiomyocyte proliferation. The role of miR-195 was unveiled by microarray analysis of RNA from ventricular samples from P1 and P10 mice [94]. miR-195 is a member of the miR-15 family and is highly upregulated between P7 to P14. Embryonic overexpression of this miRNA causes hypoplasia, septal defects, and slow-onset cardiomyopathy. Hearts transgenic for miR-195 show evidence of defective mitosis and premature cell cycle arrest. Moreover, miR-195 overexpression impairs the regenerative response of the 1-day-old mouse heart after coronary artery ligation, resulting in adult-like cardiac remodelling. On the contrary, inhibition of the miR-15 family from an early postnatal age until adulthood prevents cardiomyocyte cell cycle arrest and improves cardiac function after ischemia/reperfusion damage in adult hearts [104]. Another miRNA that is upregulated in cardiomyocytes during postnatal terminal differentiation and impairs proliferation and cardiac function when prematurely overexpressed in neonatal mice is miR-128. Inducible deletion of this miRNA extends the cardiomyocyte proliferative window by epigenetic repression of the p27 CKI and, after myocardial infarction, promotes cell cycle re-entry, thereby reducing the levels of fibrosis [105]. Finally, the levels of miR-34a, which also acts as a suppressor of cellular proliferation, increase during aging, and, after cardiac injury, only in the adult heart, suggesting a potential role for this miRNA in the loss of regenerative potential. In a consistent manner, antagonizing miR-34a expression in adult mouse hearts markedly improves cardiac repair and post-infarction remodelling, whereas increasing miR-34a expression in early postnatal hearts impairs endogenous regeneration [106].

Collectively, these studies reinforce the possibility of achieving cardiac regeneration by the exogenous activation of the cell cycle through the modulation of microRNA levels. From a translational perspective, miRNA mimics or inhibitors are appealing because of their transient action, simple chemistry and low cost.

4) **Extracellular cues that regulate cardiomyocyte proliferation.**

The above-described mechanisms that control cardiomyocyte replication by signal transduction pathways and cell cycle regulators act inside the cells but are under the control of a few extracellular cues, by which cardiomyocyte replication is regulated by events occurring in the outside environment. Deciphering how this extracellular regulation links to the intracellular pathways is crucial for both understanding why cardiomyocyte replication stops at birth and developing regenerative therapies. A few of the extracellular cues that impact on cardiomyocyte replication are discussed here.

### 2.1 Hydrodynamic pressure and sarcomere composition

The newly born heart faces a sudden increase in circulatory demand due to sustained systemic blood flow, increased blood pressure and augmented ventricular afterload. This leads to ventricular wall stress, against which the postnatal heart reacts with an extensive structural remodelling of cytoskeleton and sarcomere, by increasing both the number of fibres and contractile units and switching on the expression of some components from the foetal to the adult isoforms (reviewed in ref. [107]). The molecular mechanisms that link mechanosensing of the pressure cues to these molecular adaptations are still poorly understood. In particular, sarcomere structure could constitute a physical barrier to de-differentiation and cell cycle re-entry, as sarcomere disassembly is a prerequisite for mitotic spindle and contractile ring formation during cardiac cell division (reviewed in ref. [108]). Thus, the poor renewal rate of adult mammalian cardiomyocytes could be a trade-off for the efficient functioning of the cardiac pump. The observation that human myocardial tissue from heart failure patients implanted with left ventricular assist devices (LVADs), which provide unloading to the left ventricle, shows signs of increased proliferation [109] is consistent with these conclusions.

### 2.2 Hyperoxia, oxidative metabolism and cardiomyocyte DNA damage

During development, mammals are exposed to relatively low oxygen tension. With birth, the heart experiences a sudden increase in oxygen level due to pulmonary respiration and shunt closure. In parallel, the postnatal metabolism adapts to this context and the growing energy demand by switching from lactate anaerobic glycolysis to fatty acid oxidative phosphorylation, which ensures a more efficient production of ATP. Together, these mechanisms cause increased mitochondrial biogenesis, accumulation of reactive oxygen species (ROS) and induced oxidative stress [110]. The striking correlation between oxygen-dependent energy production and reduced cardiomyocyte proliferation argues for the possibility that the switch to oxidative metabolism could be linked to the loss of cardiac regeneration through the induction of oxidative DNA damage followed by cell-cycle arrest. Consistent with this possibility, ROS scavenging or inhibition of the DNA damage response pathways delays cell-cycle arrest of postnatal cardiomyocytes, which is instead accelerated in hyperoxia or pro-oxidant conditions [111]. Along the same line, an elegant lineage tracing experiment based on the stabilization of HIF-1α (hypoxia-inducible factor 1 alpha, a master regulator of oxygen sensing) identified a rare cardiomyocyte population that resembles proliferative neonatal cardiomyocytes [112]. These cells are small, mononucleated, with a low level of oxidative DNA damage and contribute to cardiomyocyte renewal by repressing genes coding for negative cell-cycle regulators and activating genes favouring a glycolytic metabolism [112]. Consistent with these observations, exposing animals to hypoxia reduces mitochondrial metabolism and oxidative DNA damage, which, in the context of coronary artery ligation, induces cardiac regeneration through cardiomyocyte proliferation and enhanced vascular supply [113]. Although only a small subset of cardiomyocytes upregulates HIF-1α, these findings indicate that the withdrawal of cardiomyocytes from the cell cycle may be an adaptive response to prevent the accumulation of DNA damage in mature, aerobic cells.

### 2.3 Metabolic pathways

An emerging area of investigation links specific metabolic pathways to the control of cell proliferation, including in cardiomyocytes. Neonatal mice fed with fatty-acid-deficient milk, to revert metabolic substrates to those in the foetal environment, show prolongation of the postnatal cardiomyocyte proliferative window [114]. Along the same line, the inducible cardiac deletion of pyruvate dehydrogenase kinase 4 (PDK4), which results in increased pyruvate dehydrogenase activity and glucose oxidation, decreases DNA damage and promotes cell cycle progression. After myocardial infarction, hearts from these mice show decreased dilation and remodelling [114].

The link between the activation of certain metabolic pathways and cell cycle regulation is well established in cancer cells. In particular, the muscle pyruvate kinase isoenzyme 2 (PKM2) interacts with β-catenin to enhance the activation of its downstream targets, which include c-Myc and Cyclin D1. In addition, PKM2 redirects glucose carbon flow into the pentose phosphate pathway resulting in less oxidative DNA damage [115,116]. PKM2 also exerts similar functions in cardiomyocytes. Transient administration of PKM2 modified mRNA in infarcted hearts increases cell proliferation markers, improves cardiac function and reduces scar size [117]. More recent evidence, however, has challenged this notion, by showing that the cardiac knockout of PKM2 after myocardial infarction instead exerts beneficial effects on infarct size, mitochondrial function, angiogenesis, cardiomyocyte survival and duplication [118]. Whether this discrepancy reflects a matter of gene dosage or any other specific adaptation to chronic gene deletion versus transient peak expression awaits further experimental evidence.

During ischemia, the citric acid cycle intermediate succinate accumulates from reversal of succinate dehydrogenase, which in turn is driven by fumarate overflow from purine nucleotide breakdown and partial reversal of the malate/aspartate shuttle. After reperfusion, the accumulated succinate is rapidly re-oxidized by succinate dehydrogenase (SDH), driving extensive ROS generation by reverse electron transport at mitochondrial complex I [119]. In cancer cells, a reduction in SDH activity promotes a metabolic shift into glycolysis, which drives cell division [120]. Accordingly, succinate administration in neonatal mice inhibits cardiomyocyte proliferation and regeneration, whereas SDH inhibition through malonate treatment extends the renewal window. In adult mice after myocardial infarction, SDH inhibition promotes adult cardiomyocyte proliferation, revascularization, and heart regeneration via metabolic reprogramming [121]. Collectively, these reports highlight that efficient mitochondrial oxidative metabolism is another trade-off for cardiac function at the expense of regeneration.

### 2.4 The extracellular matrix

The extracellular matrix (ECM) is a fundamental component of cardiac tissue and defines the mechano-physical properties of the cardiomyocyte extracellular milieu, as well as regulates the distribution and activation of secreted growth factors. Culturing neonatal rat cardiomyocytes on a soft matrix promotes dedifferentiation, myofibrillar disassembly and increased cell division, whereas a rigid matrix facilitates differentiation, cell cycle arrest and binucleation, with stiffness specifically affecting cardiomyocyte cytokinesis [122]. In mice, ECM composition and stiffness rapidly change between P1 and P2. In particular, a marked collagen deposition results in 50% increased ECM stiffness, which can be in part responsible for the loss of regenerative capability [123]. The molecular details on how the ECM actually regulates the cardiomyocyte cell cycle are open ground for investigation. Recent, but still scattered, evidence indicates that the cardiomyocyte sarcolemma-associated dystrophin-glycoprotein complex (DGC) could be a crucial player in the regulation of cardiomyocyte proliferation by mediating the structural relationship between the ECM and the internal actin cytoskeleton. YAP appears to be the main cardiomyocyte mechanosensor [124], while the DGC component dystroglycan 1 (Dag1) directly binds YAP to inhibit cardiomyocyte proliferation [125]. The neonatal ECM protein agrin also stimulates cardiomyocyte division through a mechanism that involves the DGC disassembly [126,127].

### 2.5 Translating experimental research towards clinical application

While a large body of information now exists on the mechanisms that regulate cardiomyocyte proliferation, translation of this information into the clinic remains challenging. This is also indirectly indicated by the relative paucity of studies that have progressed towards proving cardiac regeneration in large animals, compared to those in cells and rodents [128]. Two main hurdles that need to be overcome before cardiac regeneration becomes a clinical reality relate to the identification of effective therapeutic genes and their specific delivery into cardiomyocytes. These are reviewed in the following sections.

### 2.6 Reprogramming cardiomyocytes towards a regenerative state

One of the overarching problems in cardiac regeneration is how to find a master gene or gene cocktail that stimulates effective endogenous cardiomyocyte proliferation. It is becoming progressively clear that new cardiomyocyte formation is more complex than the regulation of cell division, and that cardiac regeneration is more complex than the formation of new cardiomyocytes. Compared to other tissues, adult cardiac fibres are formed of spatially and functionally arranged cardiomyocytes that are electrically and mechanically interconnected, with a highly hypertrophic cytoplasm enlarged in the transition from birth and adulthood to sustain increased organ mass, and containing a massive number of spatially arranged sarcomeric units and of active mitochondria. As discussed above, the physiological division of cardiomyocytes must cope with this complexity, and it does so not without molecular and spatial problems also in physiological conditions, if one has to judge from the level of polyploidy and multinucleation that most likely ensues from the problem of completing mitosis and cytokinesis respectively. In addition, the epigenetic structure of cardiomyocytes has a defined arrangement, in which several genes expressed during development are silenced by compacted chromatin conformation and cytosine methylation [26,28,129].

Stimulating the replication of cardiomyocytes requires overcoming all these layers of complexity, hence the need of “reprogramming” these cells towards a regenerative state rather than, more simply, stimulating their replication. The more effective treatments appear those that mimic the physiological signals (most likely, mechanosensing signals) that stimulate spontaneous regeneration in the neonatal mammalian heart or in the fish and amphibian hearts throughout life, or the treatments that lead to marked cell de-differentiation, such as the administration of transcription factor cocktails that transiently reprogram the cells towards an embryonic phenotype [130]. Of note, the stimulation of cardiomyocyte proliferation also appears a main driver of both the resolution of fibrosis, as fibrotic mouse hearts can still regenerate if a master regulator such as YAP is activated long after infarction [68], and the formation of new vasculature, as in all the regenerative treatments so far reported, including in large animals [101], the ratio between regenerated cardiomyocytes and vascular cells has always been found to be similar to normal.

How can we find such master regulators of complex new cardiomyocyte generation? It is possible that the identification of such genes could come through the investigation of specific pathways (for example, those leading to load mechanosensing and YAP activation), but most likely they should be sought by ex vivo and in vivo screenings for cardiomyocyte replication using genomic and ncRNA libraries with high complexity. Of note, such broad screenings have the potential to identify new factors with therapeutic function irrespective of their natural involvement in the physiological process of cardiomyocyte replication during development or in other species. In terms of activating a complex biological programme, microRNAs are of particular interest, as each of these molecules can target tens or hundreds of different cellular mRNAs. For instance, for cardiomyocyte proliferation to occur, there is a need for disassembling the sarcomere, reactivating the expression of cell cycle proteins, and reforming the centrosomes, all of which are require modulation of several different genes [128].

### 2.7 Delivering the payloads

A master problem that has affected gene therapy since its early days, and still hampers its development, is how to deliver nucleic acids efficiently into the cells and in vivo [131]. Some naturally occurring and several engineered AAV vector variants display improved cardiac selectivity. These include the AAV2i8, AAV2i8G9 and AAV-SASTG chimeras, some AAV serotype 9 variants, or vectors obtained through the screening of DNA-shuffled libraries or peptide display libraries inserted in the AAV *cap* gene [132]. Despite this improved tissue specificity, however, still very high doses of vectors are needed for AAV-based in vivo applications, in the order of 1 × 10^14^ vector genomes per kg. This high dose can end up in inflammation and both innate and acquired immunity activation [133]. This is exemplified by the toxicity observed in several recent AAV clinical trials for various neuromuscular conditions [134].

Delivery of nucleic acids using non-viral systems has advanced significantly over the last couple of decades, with a few nanoparticles that have reached clinical trials. Non-viral delivery of mRNA is currently experiencing a period of excitement after the development of lipid nanoparticles (LNPs) formed using the Stable Nucleic Acid Lipid Particle (SNALP) technology [135]. This technology has been progressively improved in the early 2000s [136,137] to become very popular after the marketing approval of patisiran for transthyretin amyloidosis in 2018 [138] and, most notably, of COVID-19 vaccines during the recent pandemic [139,140]. Once injected systemically, however, the current generation of SNALPs mainly home into the liver, and no clinically effective cell targeting system has been developed yet.

Progress in at least three areas related to nucleic acid administration would bring the cardiac gene therapy field forward, including applications for endogenous cardiac regeneration. First, the efficiency of viral vectors needs to be improved. The gold standard remains AAV vectors, but, as noted above, the efficiency of these vectors depends on the administration of large amounts of viral particles. This is required to overcome a series of stumbling blocks in the AAV vector life cycle, most notably virion trapping in unproductive endosomal routes, exit from endosomal vesicles and transport to the nucleus, and escape from the cellular DNA damage response machinery that limits conversion of single-stranded vector genome into transcription-proficient double-stranded DNA (reviewed in ref. [141]). All these processes depend on cellular proteins, which can be transiently modulated pharmacologically, or genetically using RNA inference, to improve vector efficiency.

A second long-sought improvement, which still relates to viral vectors, is the development of pharmacologically inducible promoters for controlled transgene expression. The current generation of viral vectors does not permit transcriptional regulation. This can be acceptable for applications in heart failure, in which chronic production of a therapeutic protein or ncRNA might be beneficial, but not for applications aimed to induce cardiomyocyte proliferation. Our own study with an AAV vector constitutively expressing the pro-regenerative miR-199a microRNA shows that the cardiac remuscularization that occurs in the first month from administration can be followed by hyperproliferation and the generation of fatal arrhythmias when transgene expression is not controlled [101]. Similar considerations also apply to the studies for vascular regeneration, in which the continuous expression of master angiogenesis factors, such as VEGF, can be detrimental if uncontrolled [142]. The use of promoters that can be switched on and off by pharmacological treatment [143] would solve these issues and, possibly, also permit dosing of the transgene expression.

A third holy grail of nucleic acid-based therapies for the heart is cell specific targeting. This applies to both viral and non-viral methods for gene transfer. The most efficient method for in vivo targeting is epitomized by targeting hepatocytes using *N*-acetylgalactosamine (GalNAc) trimers, which bind the cellular asialoglycoprotein receptor [144]. Both on the surface of LNPs or upon direct conjugation to ncRNAs [145], GalNac targeting permits the development of liver-specific applications upon simple systemic administration, fulfilling the demands of the Ehrlich's magic bullet. No such ligand still exists for any cell type in the heart. The field has already identified a few cardiac specific peptides through phage display panning techniques, or ligands for cardiac cell-specific receptors, or antibodies against cardiomyocyte or endothelial cell receptors (reviewed in ref. [146]). However, none of these methods appears to provide selectivity and efficacy that are comparable to that of GalNac for the liver.

In the absence of targeting, an approachable way forward for heart specific delivery is through catheter-mediated administration. Intramyocardial injections can be performed through a mini thoracotomy or during coronary bypass surgery, followed by trans-epicardial delivery. Less invasively, an intramyocardial injection can follow percutaneous intraventricular catheterisation by trans-endocardial delivery. Several catheters are already available for human application (reviewed in ref. [131]), often coupled with the possibility of pre-administration imaging for electromechanical mapping of the ventricle area to be treated. A more straightforward and less invasive administration route is intracoronary administration, which is performed for percutaneous coronary intervention (PCI; balloon angioplasty) and has already been used for a variety of clinical studies with cells and other substances [147,148]. This is particularly appealing for LNP-mediated delivery of RNA payloads after myocardial infarction, as the size of SNALPs is commonly around 100 nm in diameter, while the endothelial cell fenestrations that form in the acutely inflamed heart can reach 400–500 nm [149], thus allowing extravasation of the particles from the microcirculation into the cardiac tissue. This so called enhanced vascular permeability and retention (EPR) effect lasts approximately 48 h, which provides a sufficient time window for pro-regenerative therapies to be administered by intracoronary infusion after myocardial infarction.

## 3 Conclusions

Cardiac regeneration through the stimulation of the cardiomyocyte endogenous proliferative potential is a realistically attainable goal, provided that a series of conceptual and technical barriers are overcome. The possibility of exploiting the potential of both coding and non-coding RNAs and the development of highly processive methods for their functional screening can provide master treatments that reprogramme cardiomyocytes towards a replicative state. A major barrier remains the delivery of these regenerative nucleic acids specifically to the heart. Improvements in the design of AAV vectors, with the inclusion of pharmacologically inducible promoters, cell manipulation to transiently improve permissivity to these vectors or, alternatively, the development of non-viral methods for nucleic acid delivery that are specifically targeted to the heart are among the challenges ahead to render cardiac regenerative therapies closer to clinical application.

## References

[1] Roth G.A., Johnson C., Abajobir A., Abd-Allah F., Abera S.F., Abyu G. (2017). Global, regional, and National Burden of cardiovascular diseases for 10 causes, 1990 to 2015. J. Am. Coll. Cardiol..

[2] Roger V.L. (2013). Epidemiology of heart failure. Circ. Res..

[3] Cook C., Cole G., Asaria P., Jabbour R., Francis D.P. (2014). The annual global economic burden of heart failure. Int. J. Cardiol..

[4] Murry C.E., Reinecke H., Pabon L.M. (2006). Regeneration gaps: observations on stem cells and cardiac repair. J. Am. Coll. Cardiol..

[5] Gonzalez A., Fortuno M.A., Querejeta R., Ravassa S., Lopez B., Lopez N. (2003). Cardiomyocyte apoptosis in hypertensive cardiomyopathy. Cardiovasc. Res..

[6] Hein S., Arnon E., Kostin S., Schonburg M., Elsasser A., Polyakova V. (2003). Progression from compensated hypertrophy to failure in the pressure-overloaded human heart: structural deterioration and compensatory mechanisms. Circulation..

[7] Kyto V., Saraste A., Saukko P., Henn V., Pulkki K., Vuorinen T. (2004). Apoptotic cardiomyocyte death in fatal myocarditis. Am. J. Cardiol..

[8] Hashem S.I., Perry C.N., Bauer M., Han S., Clegg S.D., Ouyang K. (2015). Brief report: oxidative stress mediates cardiomyocyte apoptosis in a human model of Danon disease and heart failure. Stem Cells.

[9] Yamaji K., Fujimoto S., Ikeda Y., Masuda K., Nakamura S., Saito Y. (2005). Apoptotic myocardial cell death in the setting of arrhythmogenic right ventricular cardiomyopathy. Acta Cardiol..

[10] Shernan S.K. (2003). Perioperative myocardial ischemia reperfusion injury. Anesthesiol. Clin. North Am..

[11] Octavia Y., Tocchetti C.G., Gabrielson K.L., Janssens S., Crijns H.J., Moens A.L. (2012). Doxorubicin-induced cardiomyopathy: from molecular mechanisms to therapeutic strategies. J. Mol. Cell. Cardiol..

[12] Bergmann O., Bhardwaj R.D., Bernard S., Zdunek S., Barnabe-Heider F., Walsh S. (2009). Evidence for cardiomyocyte renewal in humans. Science..

[13] Soonpaa M.H., Field L.J. (1997). Assessment of cardiomyocyte DNA synthesis in normal and injured adult mouse hearts. Am. J. Phys..

[14] Senyo S.E., Steinhauser M.L., Pizzimenti C.L., Yang V.K., Cai L., Wang M. (2013). Mammalian heart renewal by pre-existing cardiomyocytes. Nature..

[15] Poss K.D., Wilson L.G., Keating M.T. (2002). Heart regeneration in zebrafish. Science..

[16] Oberpriller J.O., Oberpriller J.C. (1974). Response of the adult newt ventricle to injury. J. Exp. Zool..

[17] Poch C.M., Foo K.S., De Angelis M.T., Jennbacken K., Santamaria G., Bahr A. (2022). Migratory and anti-fibrotic programmes define the regenerative potential of human cardiac progenitors. Nat. Cell Biol..

[18] Liu Y.W., Chen B., Yang X., Fugate J.A., Kalucki F.A., Futakuchi-Tsuchida A. (2018). Human embryonic stem cell-derived cardiomyocytes restore function in infarcted hearts of non-human primates. Nat. Biotechnol..

[19] Song K., Nam Y.J., Luo X., Qi X., Tan W., Huang G.N. (2012). Heart repair by reprogramming non-myocytes with cardiac transcription factors. Nature..

[20] Qian L., Huang Y., Spencer C.I., Foley A., Vedantham V., Liu L. (2012). In vivo reprogramming of murine cardiac fibroblasts into induced cardiomyocytes. Nature..

[21] Kang M.J., Koh G.Y. (1997). Differential and dramatic changes of cyclin-dependent kinase activities in cardiomyocytes during the neonatal period. J. Mol. Cell. Cardiol..

[22] Ikenishi A., Okayama H., Iwamoto N., Yoshitome S., Tane S., Nakamura K. (2012). Cell cycle regulation in mouse heart during embryonic and postnatal stages. Develop. Growth Differ..

[23] Flink I.L., Oana S., Maitra N., Bahl J.J., Morkin E. (1998). Changes in E2F complexes containing retinoblastoma protein family members and increased cyclin-dependent kinase inhibitor activities during terminal differentiation of cardiomyocytes. J. Mol. Cell. Cardiol..

[24] Poolman R.A., Brooks G. (1998). Expressions and activities of cell cycle regulatory molecules during the transition from myocyte hyperplasia to hypertrophy. J. Mol. Cell. Cardiol..

[25] Tane S., Ikenishi A., Okayama H., Iwamoto N., Nakayama K.I., Takeuchi T. (2014). CDK inhibitors, p21(Cip1) and p27(Kip1), participate in cell cycle exit of mammalian cardiomyocytes. Biochem. Biophys. Res. Commun..

[26] Gilsbach R., Preissl S., Gruning B.A., Schnick T., Burger L., Benes V. (2014). Dynamic DNA methylation orchestrates cardiomyocyte development, maturation and disease. Nat. Commun..

[27] Quaife-Ryan G.A., Sim C.B., Ziemann M., Kaspi A., Rafehi H., Ramialison M. (2017). Multicellular transcriptional analysis of mammalian heart regeneration. Circulation..

[28] Gilsbach R., Schwaderer M., Preissl S., Gruning B.A., Kranzhofer D., Schneider P. (2018). Distinct epigenetic programs regulate cardiac myocyte development and disease in the human heart in vivo. Nat. Commun..

[29] Agah R., Kirshenbaum L.A., Abdellatif M., Truong L.D., Chakraborty S., Michael L.H. (1997). Adenoviral delivery of E2F-1 directs cell cycle reentry and p53-independent apoptosis in postmitotic adult myocardium in vivo. J. Clin. Invest..

[30] Yurkova N., Shaw J., Blackie K., Weidman D., Jayas R., Flynn B. (2008). The cell cycle factor E2F-1 activates Bnip3 and the intrinsic death pathway in ventricular myocytes. Circ. Res..

[31] Ebelt H., Zhang Y., Kampke A., Xu J., Schlitt A., Buerke M. (2008). E2F2 expression induces proliferation of terminally differentiated cardiomyocytes in vivo. Cardiovasc. Res..

[32] Sdek P., Zhao P., Wang Y., Huang C.J., Ko C.Y., Butler P.C. (2011). Rb and p130 control cell cycle gene silencing to maintain the postmitotic phenotype in cardiac myocytes. J. Cell Biol..

[33] Soonpaa M.H., Koh G.Y., Pajak L., Jing S., Wang H., Franklin M.T. (1997). Cyclin D1 overexpression promotes cardiomyocyte DNA synthesis and multinucleation in transgenic mice. J. Clin. Invest..

[34] Tane S., Kubota M., Okayama H., Ikenishi A., Yoshitome S., Iwamoto N. (2014). Repression of cyclin D1 expression is necessary for the maintenance of cell cycle exit in adult mammalian cardiomyocytes. J. Biol. Chem..

[35] Pasumarthi K.B., Nakajima H., Nakajima H.O., Soonpaa M.H., Field L.J. (2005). Targeted expression of cyclin D2 results in cardiomyocyte DNA synthesis and infarct regression in transgenic mice. Circ. Res..

[36] Toischer K., Zhu W., Hunlich M., Mohamed B.A., Khadjeh S., Reuter S.P. (2017). Cardiomyocyte proliferation prevents failure in pressure overload but not volume overload. J. Clin. Invest..

[37] Zhu W., Zhao M., Mattapally S., Chen S., Zhang J. (2018). CCND2 overexpression enhances the regenerative potency of human induced pluripotent stem cell-derived cardiomyocytes: Remuscularization of injured ventricle. Circ. Res..

[38] Zhao M., Nakada Y., Wei Y., Bian W., Chu Y., Borovjagin A.V. (2021). Cyclin D2 overexpression enhances the efficacy of human induced pluripotent stem cell-derived cardiomyocytes for myocardial repair in a swine model of myocardial infarction. Circulation..

[39] Chaudhry H.W., Dashoush N.H., Tang H., Zhang L., Wang X., Wu E.X. (2004). Cyclin A2 mediates cardiomyocyte mitosis in the postmitotic myocardium. J. Biol. Chem..

[40] Cheng R.K., Asai T., Tang H., Dashoush N.H., Kara R.J., Costa K.D. (2007). Cyclin A2 induces cardiac regeneration after myocardial infarction and prevents heart failure. Circ. Res..

[41] Woo Y.J., Panlilio C.M., Cheng R.K., Liao G.P., Atluri P., Hsu V.M. (2006). Therapeutic delivery of cyclin A2 induces myocardial regeneration and enhances cardiac function in ischemic heart failure. Circulation..

[42] Shapiro S.D., Ranjan A.K., Kawase Y., Cheng R.K., Kara R.J., Bhattacharya R. (2014). Cyclin A2 induces cardiac regeneration after myocardial infarction through cytokinesis of adult cardiomyocytes. Sci. Transl. Med..

[43] Bicknell K.A., Coxon C.H., Brooks G. (2004). Forced expression of the cyclin B1-CDC2 complex induces proliferation in adult rat cardiomyocytes. Biochem. J..

[44] Li B., Li M., Li X., Li H., Lai Y., Huang S. (2019). Sirt1-inducible deacetylation of p21 promotes cardiomyocyte proliferation. Aging (Albany NY).

[45] Di Stefano V., Giacca M., Capogrossi M.C., Crescenzi M., Martelli F. (2011). Knockdown of cyclin-dependent kinase inhibitors induces cardiomyocyte re-entry in the cell cycle. J. Biol. Chem..

[46] Mahmoud A.I., Kocabas F., Muralidhar S.A., Kimura W., Koura A.S., Thet S. (2013). Meis1 regulates postnatal cardiomyocyte cell cycle arrest. Nature..

[47] Nguyen N.U.N., Canseco D.C., Xiao F., Nakada Y., Li S., Lam N.T. (2020). A calcineurin-Hoxb13 axis regulates growth mode of mammalian cardiomyocytes. Nature..

[48] Chakraborty S., Sengupta A., Yutzey K.E. (2013). Tbx20 promotes cardiomyocyte proliferation and persistence of fetal characteristics in adult mouse hearts. J. Mol. Cell. Cardiol..

[49] Xiang F.L., Guo M., Yutzey K.E. (2016). Overexpression of Tbx20 in adult cardiomyocytes promotes proliferation and improves cardiac function after myocardial infarction. Circulation..

[50] Tang Y., Aryal S., Geng X., Zhou X., Fast V.G., Zhang J. (2022). TBX20 improves contractility and mitochondrial function during direct human cardiac reprogramming. Circulation..

[51] Mohamed T.M.A., Ang Y.S., Radzinsky E., Zhou P., Huang Y., Elfenbein A. (2018). Regulation of cell cycle to stimulate adult cardiomyocyte proliferation and cardiac regeneration. Cell..

[52] Cui M., Wang Z., Chen K., Shah A.M., Tan W., Duan L. (2020). Dynamic transcriptional responses to injury of regenerative and non-regenerative cardiomyocytes revealed by single-nucleus RNA sequencing. Dev. Cell.

[53] Li F., Wang X., Capasso J.M., Gerdes A.M. (1996). Rapid transition of cardiac myocytes from hyperplasia to hypertrophy during postnatal development. J. Mol. Cell. Cardiol..

[54] Soonpaa M.H., Kim K.K., Pajak L., Franklin M., Field L.J. (1996). Cardiomyocyte DNA synthesis and binucleation during murine development. Am. J. Phys..

[55] Alkass K., Panula J., Westman M., Wu T.D., Guerquin-Kern J.L., Bergmann O. (2015). No evidence for cardiomyocyte number expansion in preadolescent mice. Cell..

[56] Gonzalez-Rosa J.M., Sharpe M., Field D., Soonpaa M.H., Field L.J., Burns C.E. (2018). Myocardial Polyploidization creates a barrier to heart regeneration in zebrafish. Dev. Cell.

[57] Patterson M., Barske L., Van Handel B., Rau C.D., Gan P., Sharma A. (2017). Frequency of mononuclear diploid cardiomyocytes underlies natural variation in heart regeneration. Nat. Genet..

[58] Olivetti G., Cigola E., Maestri R., Corradi D., Lagrasta C., Gambert S.R. (1996). Aging, cardiac hypertrophy and ischemic cardiomyopathy do not affect the proportion of mononucleated and multinucleated myocytes in the human heart. J. Mol. Cell. Cardiol..

[59] Mollova M., Bersell K., Walsh S., Savla J., Das L.T., Park S.Y. (2013). Cardiomyocyte proliferation contributes to heart growth in young humans. Proc. Natl. Acad. Sci. U. S. A..

[60] Ovrebo J.I., Edgar B.A. (2018). Polyploidy in tissue homeostasis and regeneration. Development..

[61] Bensley J.G., De Matteo R., Harding R., Black M.J. (2016). Three-dimensional direct measurement of cardiomyocyte volume, nuclearity, and ploidy in thick histological sections. Sci. Rep..

[62] Yekelchyk M., Guenther S., Preussner J., Braun T. (2019). Mono- and multi-nucleated ventricular cardiomyocytes constitute a transcriptionally homogenous cell population. Basic Res. Cardiol..

[63] von Gise A., Lin Z., Schlegelmilch K., Honor L.B., Pan G.M., Buck J.N. (2012). YAP1, the nuclear target of hippo signaling, stimulates heart growth through cardiomyocyte proliferation but not hypertrophy. Proc. Natl. Acad. Sci. U. S. A..

[64] Xin M., Kim Y., Sutherland L.B., Qi X., McAnally J., Schwartz R.J. (2011). Regulation of insulin-like growth factor signaling by yap governs cardiomyocyte proliferation and embryonic heart size. Sci. Signal..

[65] Lin Z., von Gise A., Zhou P., Gu F., Ma Q., Jiang J. (2014). Cardiac-specific YAP activation improves cardiac function and survival in an experimental murine MI model. Circ. Res..

[66] Xin M., Kim Y., Sutherland L.B., Murakami M., Qi X., McAnally J. (2013). Hippo pathway effector yap promotes cardiac regeneration. Proc. Natl. Acad. Sci. U. S. A..

[67] Heallen T., Morikawa Y., Leach J., Tao G., Willerson J.T., Johnson R.L. (2013). Hippo signaling impedes adult heart regeneration. Development..

[68] Leach J.P., Heallen T., Zhang M., Rahmani M., Morikawa Y., Hill M.C. (2017). Hippo pathway deficiency reverses systolic heart failure after infarction. Nature..

[69] Heallen T., Zhang M., Wang J., Bonilla-Claudio M., Klysik E., Johnson R.L. (2011). Hippo pathway inhibits Wnt signaling to restrain cardiomyocyte proliferation and heart size. Science..

[70] Lin Z., Zhou P., von Gise A., Gu F., Ma Q., Chen J. (2015). Pi3kcb links hippo-YAP and PI3K-AKT signaling pathways to promote cardiomyocyte proliferation and survival. Circ. Res..

[71] Campa V.M., Gutierrez-Lanza R., Cerignoli F., Diaz-Trelles R., Nelson B., Tsuji T. (2008). Notch activates cell cycle reentry and progression in quiescent cardiomyocytes. J. Cell Biol..

[72] Collesi C., Zentilin L., Sinagra G., Giacca M. (2008). Notch1 signaling stimulates proliferation of immature cardiomyocytes. J. Cell Biol..

[73] Croquelois A., Domenighetti A.A., Nemir M., Lepore M., Rosenblatt-Velin N., Radtke F. (2008). Control of the adaptive response of the heart to stress via the Notch1 receptor pathway. J. Exp. Med..

[74] Zhao L., Borikova A.L., Ben-Yair R., Guner-Ataman B., MacRae C.A., Lee R.T. (2014). Notch signaling regulates cardiomyocyte proliferation during zebrafish heart regeneration. Proc. Natl. Acad. Sci. U. S. A..

[75] Felician G., Collesi C., Lusic M., Martinelli V., Ferro M.D., Zentilin L. (2014). Epigenetic modification at notch responsive promoters blunts efficacy of inducing notch pathway reactivation after myocardial infarction. Circ. Res..

[76] Ai D., Fu X., Wang J., Lu M.F., Chen L., Baldini A. (2007). Canonical Wnt signaling functions in second heart field to promote right ventricular growth. Proc. Natl. Acad. Sci. U. S. A..

[77] Kim S.E., Huang H., Zhao M., Zhang X., Zhang A., Semonov M.V. (2013). Wnt stabilization of beta-catenin reveals principles for morphogen receptor-scaffold assemblies. Science..

[78] Tseng A.S., Engel F.B., Keating M.T. (2006). The GSK-3 inhibitor BIO promotes proliferation in mammalian cardiomyocytes. Chem. Biol..

[79] Kerkela R., Kockeritz L., Macaulay K., Zhou J., Doble B.W., Beahm C. (2008). Deletion of GSK-3beta in mice leads to hypertrophic cardiomyopathy secondary to cardiomyoblast hyperproliferation. J. Clin. Invest..

[80] Azzolin L., Zanconato F., Bresolin S., Forcato M., Basso G., Bicciato S. (2012). Role of TAZ as mediator of Wnt signaling. Cell..

[81] Varelas X., Miller B.W., Sopko R., Song S., Gregorieff A., Fellouse F.A. (2010). The hippo pathway regulates Wnt/beta-catenin signaling. Dev. Cell.

[82] Liang J., Slingerland J.M. (2014). Multiple roles of the PI3K/PKB (Akt) pathway in cell cycle progression. Cell Cycle.

[83] Cross D.A.E., Alessi D.R., Cohen P., Andjelkovich M., Hemmings B.A. (1995). Inhibition of glycogen synthase kinase-3 by insulin mediated by protein kinase B. Nature..

[84] D’Uva G., Aharonov A., Lauriola M., Kain D., Yahalom-Ronen Y., Carvalho S. (2015). ERBB2 triggers mammalian heart regeneration by promoting cardiomyocyte dedifferentiation and proliferation. Nat. Cell Biol..

[85] Bersell K., Arab S., Haring B., Kuhn B. (2009). Neuregulin1/ErbB4 signaling induces cardiomyocyte proliferation and repair of heart injury. Cell..

[86] Chen Z., Xie J., Hao H., Lin H., Wang L., Zhang Y. (2017). Ablation of periostin inhibits post-infarction myocardial regeneration in neonatal mice mediated by the phosphatidylinositol 3 kinase/glycogen synthase kinase 3beta/cyclin D1 signalling pathway. Cardiovasc. Res..

[87] Kuhn B., del Monte F., Hajjar R.J., Chang Y.S., Lebeche D., Arab S. (2007). Periostin induces proliferation of differentiated cardiomyocytes and promotes cardiac repair. Nat. Med..

[88] Engel F.B., Schebesta M., Duong M.T., Lu G., Ren S., Madwed J.B. (2005). p38 MAP kinase inhibition enables proliferation of adult mammalian cardiomyocytes. Genes Dev..

[89] Engel F.B., Hsieh P.C., Lee R.T., Keating M.T. (2006). FGF1/p38 MAP kinase inhibitor therapy induces cardiomyocyte mitosis, reduces scarring, and rescues function after myocardial infarction. Proc. Natl. Acad. Sci. U. S. A..

[90] Li Q., Li B., Wang X., Leri A., Jana K.P., Liu Y. (1997). Overexpression of insulin-like growth factor-1 in mice protects from myocyte death after infarction, attenuating ventricular dilation, wall stress, and cardiac hypertrophy. J. Clin. Invest..

[91] Wei K., Serpooshan V., Hurtado C., Diez-Cuñado M., Zhao M., Maruyama S. (2015). Epicardial FSTL1 reconstitution regenerates the adult mammalian heart. Nature..

[92] Altekoester A.K., Harvey R.P. (2015). Bioengineered FSTL1 patches restore cardiac function following myocardial infarction. Trends Mol. Med..

[93] Magadum A., Singh N., Kurian A.A., Sharkar M.T.K., Chepurko E., Zangi L. (2018). Ablation of a single N-glycosylation site in human FSTL 1 induces cardiomyocyte proliferation and cardiac regeneration. Mol. Ther. Nucleic Acids..

[94] Porrello E.R., Johnson B.A., Aurora A.B., Simpson E., Nam Y.J., Matkovich S.J. (2011). MiR-15 family regulates postnatal mitotic arrest of cardiomyocytes. Circ. Res..

[95] Eulalio A., Mano M., Dal Ferro M., Zentilin L., Sinagra G., Zacchigna S. (2012). Functional screening identifies miRNAs inducing cardiac regeneration. Nature..

[96] Diez-Cunado M., Wei K., Bushway P.J., Maurya M.R., Perera R., Subramaniam S. (2018). miRNAs that induce human cardiomyocyte proliferation converge on the hippo pathway. Cell Rep..

[97] Chen J., Huang Z.P., Seok H.Y., Ding J., Kataoka M., Zhang Z. (2013). Mir-17-92 cluster is required for and sufficient to induce cardiomyocyte proliferation in postnatal and adult hearts. Circ. Res..

[98] Gao F., Kataoka M., Liu N., Liang T., Huang Z.P., Gu F. (2019). Therapeutic role of miR-19a/19b in cardiac regeneration and protection from myocardial infarction. Nat. Commun..

[99] Tian Y., Liu Y., Wang T., Zhou N., Kong J., Chen L. (2015). A microRNA-hippo pathway that promotes cardiomyocyte proliferation and cardiac regeneration in mice. Sci. Transl. Med..

[100] Lesizza P., Prosdocimo G., Martinelli V., Sinagra G., Zacchigna S., Giacca M. (2017). Single-dose Intracardiac injection of pro-regenerative MicroRNAs improves cardiac function after myocardial infarction. Circ. Res..

[101] Gabisonia K., Prosdocimo G., Aquaro G.D., Carlucci L., Zentilin L., Secco I. (2019). MicroRNA therapy stimulates uncontrolled cardiac repair after myocardial infarction in pigs. Nature..

[102] Zhen L., Zhao Q., Lu J., Deng S., Xu Z., Zhang L. (2020). miR-301a-PTEN-AKT signaling induces cardiomyocyte proliferation and promotes cardiac repair post-MI. Mol. Ther. Nucleic Acids..

[103] Borden A., Kurian J., Nickoloff E., Yang Y., Troupes C.D., Ibetti J. (2019). Transient introduction of miR-294 in the heart promotes cardiomyocyte cell cycle reentry after injury. Circ. Res..

[104] Porrello E.R., Mahmoud A.I., Simpson E., Johnson B.A., Grinsfelder D., Canseco D. (2013). Regulation of neonatal and adult mammalian heart regeneration by the miR-15 family. Proc. Natl. Acad. Sci. U. S. A..

[105] Huang W., Feng Y., Liang J., Yu H., Wang C., Wang B. (2018). Loss of microRNA-128 promotes cardiomyocyte proliferation and heart regeneration. Nat. Commun..

[106] Yang Y., Cheng H.W., Qiu Y., Dupee D., Noonan M., Lin Y.D. (2015). MicroRNA-34a plays a key role in cardiac repair and regeneration following myocardial infarction. Circ. Res..

[107] Ahmed R.E., Tokuyama T., Anzai T., Chanthra N., Uosaki H. (1864). Sarcomere maturation: function acquisition, molecular mechanism, and interplay with other organelles. Philos. Trans. R. Soc. Lond. Ser. B Biol. Sci..

[108] Ali H., Braga L., Giacca M. (2020). Cardiac regeneration and remodelling of the cardiomyocyte cytoarchitecture. FEBS J..

[109] Canseco D.C., Kimura W., Garg S., Mukherjee S., Bhattacharya S., Abdisalaam S. (2015). Human ventricular unloading induces cardiomyocyte proliferation. J. Am. Coll. Cardiol..

[110] Ishihara T., Ban-Ishihara R., Maeda M., Matsunaga Y., Ichimura A., Kyogoku S. (2015). Dynamics of mitochondrial DNA nucleoids regulated by mitochondrial fission is essential for maintenance of homogeneously active mitochondria during neonatal heart development. Mol. Cell. Biol..

[111] Puente B.N., Kimura W., Muralidhar S.A., Moon J., Amatruda J.F., Phelps K.L. (2014). The oxygen-rich postnatal environment induces cardiomyocyte cell-cycle arrest through DNA damage response. Cell..

[112] Kimura W., Xiao F., Canseco D.C., Muralidhar S., Thet S., Zhang H.M. (2015). Hypoxia fate mapping identifies cycling cardiomyocytes in the adult heart. Nature..

[113] Nakada Y., Canseco D.C., Thet S., Abdisalaam S., Asaithamby A., Santos C.X. (2017). Hypoxia induces heart regeneration in adult mice. Nature..

[114] Cardoso A.C., Lam N.T., Savla J.J., Nakada Y., Pereira A.H.M., Elnwasany A. (2020). Mitochondrial substrate utilization regulates cardiomyocyte cell cycle progression. Nat. Metab..

[115] Yang W., Xia Y., Ji H., Zheng Y., Liang J., Huang W. (2011). Nuclear PKM2 regulates beta-catenin transactivation upon EGFR activation. Nature..

[116] Kumar B., Bamezai R.N. (2015). Moderate DNA damage promotes metabolic flux into PPP via PKM2 Y-105 phosphorylation: a feature that favours cancer cells. Mol. Biol. Rep..

[117] Magadum A., Singh N., Kurian A.A., Munir I., Mehmood T., Brown K. (2020). Pkm2 regulates cardiomyocyte cell cycle and promotes cardiac regeneration. Circulation..

[118] Hauck L., Dadson K., Chauhan S., Grothe D., Billia F. (2021). Inhibiting the Pkm2/b-catenin axis drives in vivo replication of adult cardiomyocytes following experimental MI. Cell Death Differ..

[119] Chouchani E.T., Pell V.R., Gaude E., Aksentijevic D., Sundier S.Y., Robb E.L. (2014). Ischaemic accumulation of succinate controls reperfusion injury through mitochondrial ROS. Nature..

[120] Gottlieb E., Tomlinson I.P. (2005). Mitochondrial tumour suppressors: a genetic and biochemical update. Nat. Rev. Cancer.

[121] Bae J., Salamon R.J., Brandt E.B., Paltzer W.G., Zhang Z., Britt E.C. (2021). Malonate promotes adult cardiomyocyte proliferation and heart regeneration. Circulation..

[122] Yahalom-Ronen Y., Rajchman D., Sarig R., Geiger B., Tzahor E. (2015). Reduced matrix rigidity promotes neonatal cardiomyocyte dedifferentiation, proliferation and clonal expansion. Elife..

[123] Notari M., Ventura-Rubio A., Bedford-Guaus S.J., Jorba I., Mulero L., Navajas D. (2018). The local microenvironment limits the regenerative potential of the mouse neonatal heart. Sci. Adv..

[124] Nardone G., Oliver-De La Cruz J., Vrbsky J., Martini C., Pribyl J., Skladal P. (2017). YAP regulates cell mechanics by controlling focal adhesion assembly. Nat. Commun..

[125] Morikawa Y., Heallen T., Leach J., Xiao Y., Martin J.F. (2017). Dystrophin-glycoprotein complex sequesters yap to inhibit cardiomyocyte proliferation. Nature..

[126] Bassat E., Mutlak Y.E., Genzelinakh A., Shadrin I.Y., Baruch Umansky K., Yifa O. (2017). The extracellular matrix protein agrin promotes heart regeneration in mice. Nature..

[127] Baehr A., Umansky K.B., Bassat E., Jurisch V., Klett K., Bozoglu T. (2020). Agrin promotes coordinated therapeutic processes leading to improved cardiac repair in pigs. Circulation..

[128] Braga L., Ali H., Secco I., Giacca M. (2021). Non-coding RNA therapeutics for cardiac regeneration. Cardiovasc. Res..

[129] Preissl S., Schwaderer M., Raulf A., Hesse M., Gruning B.A., Kobele C. (2015). Deciphering the epigenetic code of cardiac myocyte transcription. Circ. Res..

[130] Chen Y., Luttmann F.F., Schoger E., Scholer H.R., Zelarayan L.C., Kim K.P. (2021). Reversible reprogramming of cardiomyocytes to a fetal state drives heart regeneration in mice. Science..

[131] Cannata A., Ali H., Sinagra G., Giacca M. (2020). Gene therapy for the heart lessons learned and future perspectives. Circ. Res..

[132] Grimm D., Buning H. (2017). Small but increasingly mighty: latest advances in AAV vector research, design, and evolution. Hum. Gene Ther..

[133] Ertl H.C.J. (2022). Immunogenicity and toxicity of AAV gene therapy. Front. Immunol..

[134] Rapti K., Grimm D. (2021). Adeno-associated viruses (AAV) and host immunity - a race between the hare and the hedgehog. Front. Immunol..

[135] Kulkarni J.A., Cullis P.R., van der Meel R. (2018). Lipid nanoparticles enabling gene therapies: from concepts to clinical utility. Nucleic Acid Ther..

[136] MacLachlan I., Cullis P., Graham R.W. (1999). Progress towards a synthetic virus for systemic gene therapy. Curr. Opin. Mol. Ther..

[137] Heyes J., Palmer L., Bremner K., MacLachlan I. (2005). Cationic lipid saturation influences intracellular delivery of encapsulated nucleic acids. J. Control. Release.

[138] Adams D., Gonzalez-Duarte A., O’Riordan W.D., Yang C.C., Ueda M., Kristen A.V. (2018). Patisiran, an RNAi therapeutic, for hereditary transthyretin amyloidosis. N. Engl. J. Med..

[139] Baden L.R., El Sahly H.M., Essink B., Kotloff K., Frey S., Novak R. (2021). Efficacy and safety of the mRNA-1273 SARS-CoV-2 vaccine. N. Engl. J. Med..

[140] Polack F.P., Thomas S.J., Kitchin N., Absalon J., Gurtman A., Lockhart S. (2020). Safety and efficacy of the BNT162b2 mRNA Covid-19 vaccine. N. Engl. J. Med..

[141] Zacchigna S., Zentilin L., Giacca M. (2014). Adeno-associated virus vectors as therapeutic and investigational tools in the cardiovascular system. Circ. Res..

[142] Zacchigna S., Tasciotti E., Kusmic C., Arsic N., Sorace O., Marini C. (2007). In vivo imaging shows abnormal function of vascular endothelial growth factor-induced vasculature. Hum. Gene Ther..

[143] Tafuro S., Ayuso E., Zacchigna S., Zentilin L., Moimas S., Dore F. (2009). Inducible adeno-associated virus vectors promote functional angiogenesis in adult organisms via regulated vascular endothelial growth factor expression. Cardiovasc. Res..

[144] Springer A.D., Dowdy S.F. (2018). GalNAc-siRNA conjugates: leading the way for delivery of RNAi therapeutics. Nucleic Acid Ther..

[145] Huang Y. (2017). Preclinical and clinical advances of GalNAc-decorated nucleic acid therapeutics. Mol. Ther. Nucleic Acids..

[146] Shah A.M., Giacca M. (2022). Small non-coding RNA therapeutics for cardiovascular disease. Eur. Heart J..

[147] Chen S., Liu Z., Tian N., Zhang J., Yei F., Duan B. (2006). Intracoronary transplantation of autologous bone marrow mesenchymal stem cells for ischemic cardiomyopathy due to isolated chronic occluded left anterior descending artery. J. Invasive Cardiol..

[148] Sheng C.C., Zhou L., Hao J. (2013). Current stem cell delivery methods for myocardial repair. Biomed. Res. Int..

[149] Fang J., Islam W., Maeda H. (2020). Exploiting the dynamics of the EPR effect and strategies to improve the therapeutic effects of nanomedicines by using EPR effect enhancers. Adv. Drug Deliv. Rev..

